# Effect of phytochemical-filled microcapsules with antifungal activity on material properties and dimensional accuracy of denture base resin for three-dimensional printing

**DOI:** 10.1186/s12903-022-02216-z

**Published:** 2022-05-13

**Authors:** Sol Jeon, Ye-Hyeon Jo, Hyung-In Yoon, Jung-Suk Han

**Affiliations:** 1 Department of Prosthodontics, School of Dentistry and Dental Research Institute, Seoul National University, 101, Daehak-ro, Jongro-gu, Seoul, 03080 Republic of Korea; 2 Dental Research Institute, Seoul National University School of Dentistry, Seoul, South Korea

**Keywords:** Digital light processing, Denture base, Phytochemical, Microcapsule, Dimensional accuracy, Flexural strength

## Abstract

**Background:**

Studies on the material properties and dimensional accuracy of three-dimensionally (3D) printed denture base containing microcapsules with antifungal phytochemicals are lacking.

**Methods:**

Two types of phytochemicals (phytoncide A and B) with antifungal activity were microencapsulated. The 3D-printed denture base specimens with minimum and maximum effective concentrations of microcapsules (6 and 8 wt% for phytoncide A; 15 and 25 wt% for phytoncide B) were prepared. The morphological changes of *C. albicans* on 3D-printed denture base with microcapsules was microscopically observed. The degree of conversion of 3D-printed denture base with microcapsules investigated. The microhardness and flexural strength values were also measured to evaluate the mechanical properties of 3D-printed denture bases. The dimensional accuracy (trueness) of the specimens with microcapsules was measured as root-mean-square values (RMS) for the whole, upper, and side surfaces of the specimens as well as their total height. For the degree of conversion, microhardness, and flexural strength values, the Kruskal–Wallis analysis and a post-hoc comparison using Mann–Whitney U test was performed. For the analysis of trueness (RMS), the one-way analysis of variance and a post-hoc comparison using Tukey’s method was conducted (α = 0.05).

**Results:**

At both maximum and minimum effective concentrations of microcapsules, cell surface disruption or membrane breakdown of fungal cells were observed in the specimens. The groups with microcapsules (both phytoncide A- and B-filled) showed significantly lower microhardness and elastic modulus values than the control group (all, P = 0.001). For the trueness, all the RMS values of the whole, upper, and side surfaces of the specimens with microcapsules were less than 100 µm, although significantly higher than those without (all, P = 0.001). The mean flexural strength values of the groups with phytoncide A-filled microcapsule were higher than 65 MPa, not statistically different from that of the control group (all, P > 0.05). However, the groups with phytoncide B-filled microcapsules showed significantly lower values than the control (all, P = 0.001).

**Conclusions:**

Within the limitations of this in-vitro study, the 3D-printed denture base containing 6 wt% of phytoncide A-filled microcapsules was clinically acceptable in terms of antifungal activity, dimensional accuracy, and flexural strength.

## Introduction

The technology for producing removable dentures has been developed with advances in computer-aided design/computer-aided manufacturing (CAD/CAM), particularly using scanners and three-dimensional (3D) printers [1–3]. Low material consumption, simplified laboratory steps, and faster manufacturing processes are the main advantages of 3D-printed removable dentures [4–6]. Several studies have reported that the accuracy of the intaglio surface of the 3D-printed denture base is clinically acceptable [7–9]. Among various techniques of 3D printing, the digital light processing (DLP) was reported to show high manufacturing speed and achieve higher resolution by ultra-fast shifting and integral projecting [10]. Hwang et al. [8] reported that the trueness of the maxillary complete denture base produced by digital light processing (DLP) was superior to that produced by milling or conventional workflow, exhibiting tissue surface adaptation within 100 μm. Yoon et al. [7] suggested that the mandibular complete denture bases fabricated by DLP showed a clinically acceptable range of adaptation and accuracy. In a clinical study, Christache et al. [11] reported that patients using complete dentures produced by the additive method showed high satisfaction. Yoon et al. [12] reported that the denture base fabricated by the DLP showed clinically acceptable tissue surface adaptation. In addition, the strength of the denture base material is also essential for the success of denture treatment, as the external loading force is exerted on the dentures during mastication [13, 14]. The flexural strength of the 3D-printed denture base was reported to be higher than 60 MPa, satisfying the requirements for clinical use [15, 16]. Denture base materials must have adequate hardness to resist surface wear, which may affect microbial accumulation when using complete dentures [17, 18]. However, some denture base materials fabricated by 3D printing showed lower hardness than those fabricated by conventional method [15].

The prevalence of denture stomatitis in denture-wearing patients ranges from 15 to 70%. Poor oral hygiene, persistent denture wearing, and pathogenic fungal infections could be etiological factors [19]. *Candida albicans* is an opportunistic infectious pathogen in the oral cavity that forms a biofilm on the denture surface, causing inflammation of the oral mucosa [20, 21]. The possibility of adhesion of *C. albicans* on the denture surface was reported to be higher in 3D printing than in conventional methods, which may increase the possibility of Candida-associated denture stomatitis [22]. Various studies have been conducted to add antimicrobial activity to the denture base resin material, including the use of monomers or copolymers with antimicrobial effects [23, 24], phytochemical components [25, 26], and surface coating [27]. The application of antifungal agents or antimicrobial monomers was effective in reducing biofilm formation by microorganisms; however, showed side effects such as cytotoxicity and bacterial resistance [28, 29]. In contrast, the phytochemical components from natural organic extracts are released quickly, showing antimicrobial activity with small side effects [26]. The rapid release of natural organic extracts can be solved by microencapsulation of phytochemical components as core materials [30, 31]. Among the various phytochemical components, phytoncide has antimicrobial effects on bacteria and fungi [32]. Lee et al. [33] reported that denture base resins containing phytoncide showed antifungal effects. Additionally, An et al. [26] reported that a complete denture with an antifungal effect can be manufactured by mixing microcapsules containing phytoncide with denture resin to solve the rapid release of phytochemical agents. Recently, the in-vitro research by Jeon et al. [34] reported that denture base resin with antifungal activities could be manufactured using micro-encapsulation of phytochemicals and 3D printing. The researchers evaluated the antifungal effect and cytotoxicity of the DLP-generated denture base with phytoncide-filled microcapsules. At the specific microcapsule concentrations, the denture bases with phytoncide-filled microcapsules showed antifungal activities against *C. albicans*, persistent for 4 weeks [34]. No cytotoxicity to human gingival fibroblasts was reported. Furthermore, incorporation of microcapsules into denture resin did not significantly increase the surface roughness of the 3D-printed objects.

To the best of our knowledge, there are no studies on the effect of microcapsules containing antifungal phytochemicals on the material properties and dimensional accuracy of denture base resin for 3D printing. Therefore, the aim of this research is to investigate the material properties (degree of conversion, microhardness and flexural strength) and dimensional accuracy (trueness) of the 3D-printed denture bases containing phytoncide-filled microcapsules with antifungal activities at maximum and minimum effective concentrations. The null hypothesis was that there were no differences in the material properties and dimensional accuracy of 3D-printed denture bases, regardless of the presence of phytoncide-filled microcapsules.

## Methods

The microcapsules containing two different types of phytoncide oil extract, effectively known as antifungal activity to *C. albicans* [26]*,* were mixed with a 3D printable denture base resin (NextDent Denture 3D+ ; Vertex Dental BV, Soesterberg, Netherlands). Phytoncide oil type A, extracted from *Pinus densiflora* (Cleandiox; Gyeonggi-do, Korea), and phytoncide oil type B, extracted from *Chamaecyparis obtusa* (Cleandiox; Gyeonggi-do, Korea) were used for micro-encapsulation. Each phytoncide oil was microencapsulated as follows: distilled water was added as a stabilizer to the styrene-maleic anhydride copolymer solution and the phytoncide oil was added in the form of droplets. Thereafter, the melamine urea–formaldehyde prepolymer was dissolved to form a capsule wall. After stirring at 60 °C in a pH 3–5 environment, the solution was aged and dried. The detailed information of the microencapsulation process used in this study was previously reported [34]. Based on the findings of the in-vitro study by Jeon et al. [34], the maximum effective concentration of microcapsules in the mixture (3D-printable denture base resin, phytoncide-filled microcapsules, and dispersant) showing antifungal activity were set as 8 wt% for phytoncide type A and 25 wt% for type B. In addition, the minimum effective concentration of microcapsules in the mixture with antifungal activity were set as 6 wt% for phytoncide type A and 15 wt% for phytoncide type B for this study. A dispersant (DISPERBYK-111; BYK, Chemie, Germany) was added to the mixture to evenly disperse the capsules and added with a condition of 20 wt% of the mass of the incorporated microcapsules. No microcapsule and dispersant but only the 3D-printable denture base resin was included in the specimen of control group. Therefore, four different groups (A 6 wt%, A 8 wt%, B 15 wt%, and B 25 wt%) of 3D-printable resin mixtures and the control group of denture resin with no microcapsules were prepared.

A disc shape with 15 mm-diameter and 5 mm-height was virtually designed (TinkerCAD, Autodesk, Montreal, Quebec, Canada) and stored in a standard tessellation language (STL) file format. Using the CAD file, the denture resin disc specimens were manufactured by a DLP printer (Max UV; Asiga, Sydney, Australia) and 3D printable resin with the following parameters: layer thickness of 50 µm, light intensity of 7.50 mW/cm^2^, exposure time of 2 s, build angle of 0°, and light source wavelength of 385 nm. The supports were automatically positioned and then placed using the software (Asiga Composer; Asiga, Sydney, Australia). After each printing, the specimens were washed for 10 min using a washing machine (Form wash; Formlabs, Somerville, USA) containing ethanol, and a curing machine (Cure M U102H; Graphy, Seoul, Korea) was used to perform post-curing for 5 min according to the manufacturer’s recommendations. All the evaluations of 3D-printed resin discs were conducted on the surfaces opposite to the areas with support structures. First, to check the antifungal activity of the 3D-printed resin discs with phytoncide-filled microcapsules, the morphological change of *C. albicans* on the surface of each disc was microscopically observed (n = 5 per each group). The specimens with *C. albicans* (ATCC 10,231), obtained from the Korean Collection of Microorganisms, were incubated for 24 h, washed twice with Dulbecco’s phosphate-buffered saline (DPBS), and fixed in 2.5% (v/v) glutaraldehyde (Sigma-Aldrich; St. Louis, MO, USA) for 2 h. After washing twice using DBPS, they were fixed in 1% osmium tetroxide for 30 min. After dehydration of the sample in an ethanol solution, each sample was sputtered with platinum, and the surface of the coated specimen was observed using field emission scanning electron microscopy (FE-SEM, Apreo S; Thermo Fisher Scientific, Waltham, MA, USA) at a voltage of 10 kV. Second, to investigate the effect of phytoncide-filled microcapsules on the degree of conversion, the infrared spectra of 3D-printed denture resin discs (n = 5 per each group) with microcapsules, as well as the uncured resin materials before 3D printing, were recorded using a Fourier transform infrared spectrometer (FT-IR, TENSOR27, Bruker, Germany) between 4000 and 400 cm^−1^ in the transmittance mode. For the specimen of each group, the measurement was repeated for three times and the degree of conversion (%) of the denture base resin for 3D printing was calculated using the following equation:

$${\text{Degree }}\,{\text{of}}\, {\text{Conversion}} \left( {DC} \right) = \left\{ {1 - \frac{{Abs_{aliphatic} \left( {polymer} \right)/Abs_{aromatic} \left( {polymer} \right)}}{{Abs_{aliphatic} \left( {monomer} \right)/Abs_{aromatic} \left( {monomer} \right)}}} \right\} \times 100$$

where *Abs*_*aliphatic*_ is the absorbance peak areas of the aliphatic bond of the material measured from the peak at 1638 cm^−1^ as the intensity of the aliphatic bond and *Abs*_*aromatic*_ is the absorbance peak areas of the aromatic bond of the material measured from the peak at 1610 cm^−1^ as the intensity of the aromatic bond. Third, to evaluate the effect of microcapsule on the microhardness of the 3D-printed denture base resin discs, the surfaces of the specimens (n = 5 per each group) were further polished to allow for better identification of indentation using a polishing machine (Phoenix, Bühler, Düsseldorf, Germany) at 150 rpm with 4000 grit SiC papers. The microhardness (H, GPa) and elastic modulus (E, GPa) were determined by probing the pyramidal Berkovich diamond tip of a nanoindenter (Ubi-1, Hysitron, Minneapolis, MN, USA). The nanoindenter was operated by load control up to a peak load of 1000 μN for contact depths above 500 nm.

A composite piled tower of four cylinders with different diameters was virtually designed as a test specimen (reference CAD data) to evaluate the effect of microcapsule on the dimensional accuracy of the 3D-printed denture base. Considering the characteristics of the light source on the accuracy, the cylinders with different diameters were designed to simulate horizontal reproduction in x-, y- axis, while the vertically piled tower was to test the resolution along the z- axis [35]. The tower-shaped specimens for each group (n = 10) were fabricated using the same manufacturing protocols set above. After post-processing, all the specimens were digitally scanned as STL files using a laboratory scanner (Medit T500; Medit Corp, Seoul, Korea) in standard mode. To evaluate the dimensional accuracy, the 3D-printed tower-shaped specimens from the groups with microcapsules were compared with those from the control group in terms of the trueness, which is defined as the closeness of agreement between the expectation of a measurement result and the true value [36]. Each scanned STL file was superimposed on the reference CAD data using the iterative closest point algorithm and best-fit alignment, by the inspection software (Geomagic Control X; 3D Systems, Rock Hill, SC, USA). The reference CAD data of the tower-shaped specimen was divided into four analytic regions as follows: (1) whole surfaces except for the bottom of the cylinders, (2) upper surfaces of the cylinders, (3) side surfaces of the cylinders, and (4) total height of the cylinders. The bottom surface, where the supports were attached, was excluded to minimize possible errors in the superimposition analysis. Except the total height (mm) measured using the tools of the inspection software, the root-mean-square value (RMS, µm) was calculated for each analysis (whole, upper, and side surface) using the formula below:

$${\text{RMS}} = \frac{{\sqrt {\mathop \sum \nolimits_{i = 1}^{n} \left( {X_{1,i} - X_{2,i} } \right)^{2} } }}{\sqrt n }$$

where *X*_*1, i*_ is the measuring point *i* on the reference data; *X*_*2, i*_ is the measuring point *i* on the scan data; *n* is the total number of measuring points. A 3D color deviation map was also displayed for each superimposition analysis, with a nominal deviation of ± 50 mm and a critical deviation of ± 500 mm.

To measure the flexural strength of the 3D-printed denture base with phytoncide-filled microcapsules in accordance with ISO 20975-1: 2013 [37], the bar-shaped specimens (65 × 10 × 3.3 ± 0.2 mm) were virtually designed as STL files and fabricated for each group (n = 10) from the 3D printable resin materials with the same printing parameters and post-processing protocols set above. For the control group, the specimens with identical dimension and shapes were produced with the resin with no microcapsules. The flexural strength value (MPa) of each denture resin bar-shaped specimen was measured using a three-point bending test apparatus with a universal testing machine (Instron 8871; Instron, Canton, MA) at a crosshead speed of 5 mm/min. The flexural strength (FS) and flexural modulus (E) were computed using the following equation:

$${\text{FS}} = \frac{3Pl}{{2bd^{2} }},\,E = \frac{{Fl^{3} }}{{4ybd^{3} }}$$

where *P* is the maximum load before fracture, *l* is the distance between the supports (50 mm), *b* is the width of the specimen, *d* is the thickness of the specimen, and *y* is the deflection due to the load *F* applied at the middle of the specimen. The fractured surfaces of the tested specimens were platinum-coated and observed using FE-SEM (Apreo S, Thermo Fisher Scientific, MA, USA).

For the degree of conversion, microhardness values, and flexural strength values, the normality test of the data was not satisfied by the Shapiro–Wilk test and the Kruskal–Wallis test was performed. A post hoc multiple comparison test was performed using the Mann–Whitney U test, adjusted by the Bonferroni’s method. As the RMS data measured at the whole, upper, side surfaces and total height of the tower-shaped specimens passed the Shapiro–Wilk test for normality and Levene test of equal variance, the one-way analysis of variance was conducted. A post-hoc multiple comparison was conducted using the Tukey’s method. All statistical analyses were performed (IBM SPSS Statistics, v25.0; IBM Corp, USA) with a level of significance (α) of 0.05.

## Results

**Fig. 1:**
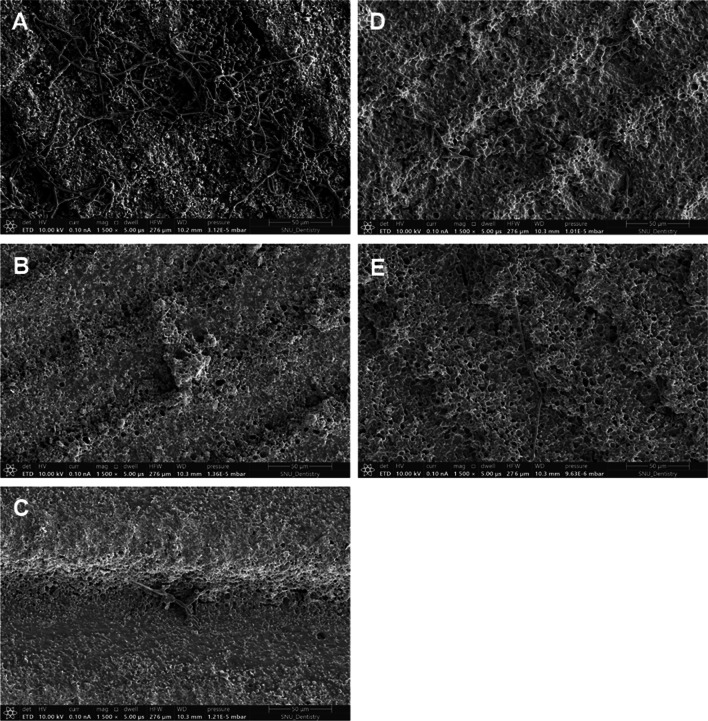
Microscopic images (magnification: × 1500) of *C. albicans* adhesion for 3D-printed denture base specimens containing phytochemical-filled microcapsules. **A** Control (no microcapsule); **B** and **C** Phytoncide A-filled, 6 wt% and 8 wt%, respectively; **D** and **E** Phytoncide B-filled, 15 wt% and 25wt%, respectively. (Fig. 1). Compared with the control group, distinctive morphological changes such as cell surface disruption or membrane breakdown in the fungal cells were evident from **B** to **E**

**Fig. 2:**
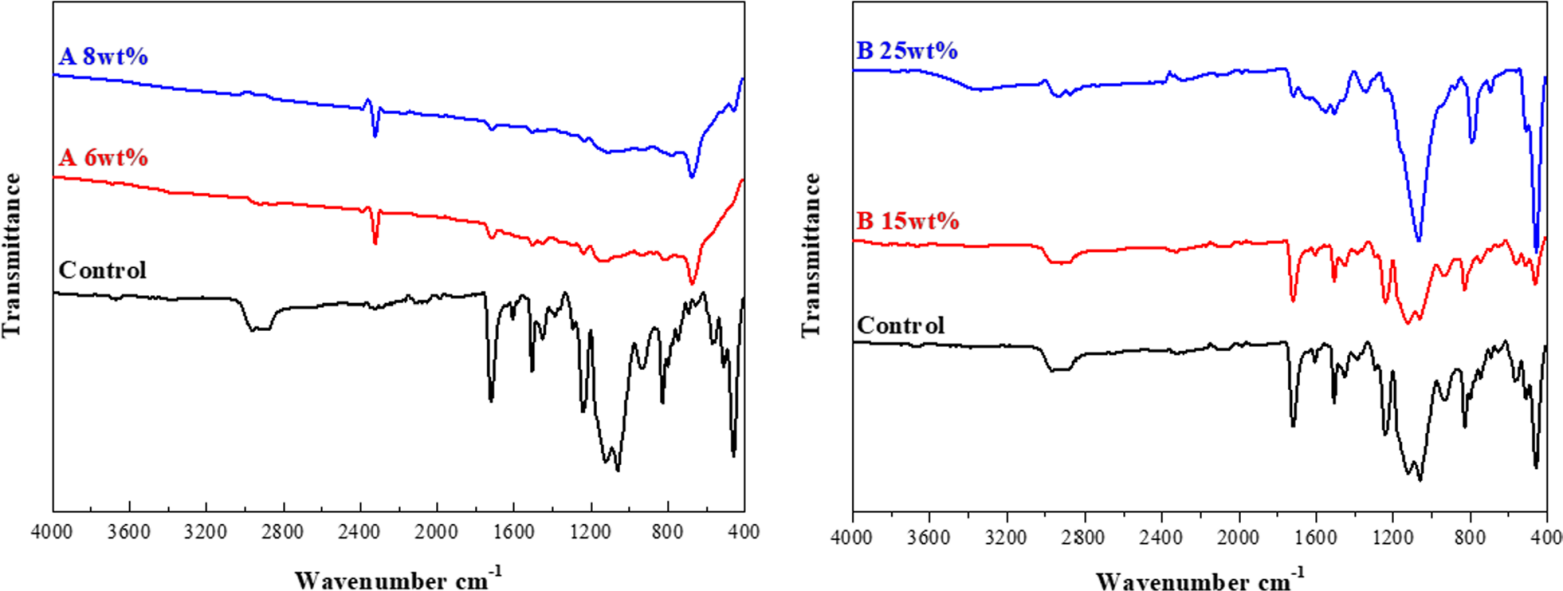
Infrared spectra of 3D-printed denture base resin discs with phytoncide A-filled and B-filled microcapsules are displayed. **A**: phytoncide A-filled microcapsule, 6 and 8 wt%; **B**: phytoncide B-filled microcapsule, 15 and 25 wt%. Spectra of discs with no microcapsules (control group) are also displayed

**Fig. 3:**
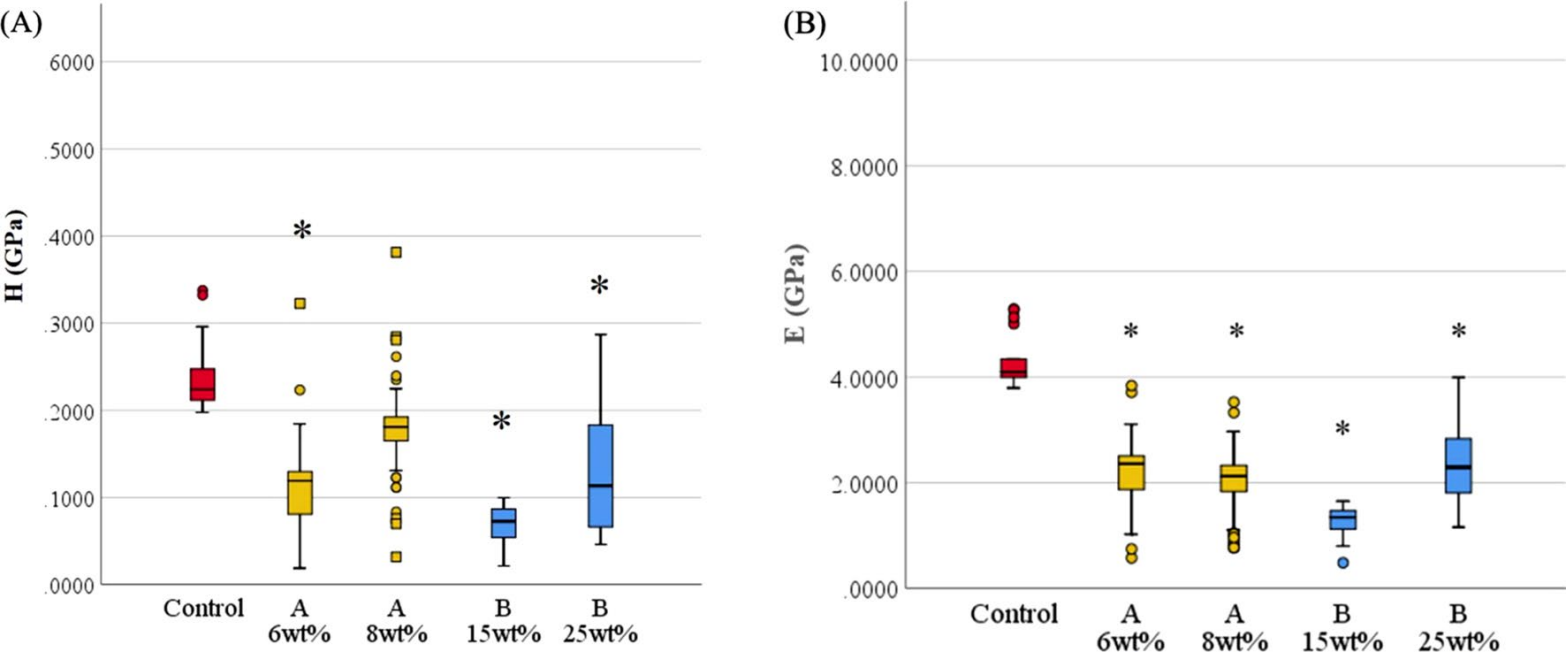
Microhardness (H, Gpa) and elastic modulus (E, GPa) values of 3D-printed denture resin specimens. **A** Microhardness; **B** Elastic modulus. Statistically significant differences among groups were marked with asterisks (P < 0.05). Control: no microcapsule; **A**: phytoncide A-filled microcapsule, 6 and 8 wt%; **B**: phytoncide B-filled microcapsule, 15 and 25 wt%

The microscopic examination revealed that adding phytoncide A-filled and B-filled microcapsules to 3D-printed denture base resin reduced the adhesion of *C. albicans*, showing distinctive morphological changes such as cell surface disruption or membrane breakdown in the fungal cells (Fig. 1). Compared with the control group, the decrease of fungal cell adhesion as well as the breakdown of cell structure was evident on the specimens at the minimum effective concentration of phytoncide-filled microcapsules (A 6 wt% and B 15 wt%). At the maximum concentration of microcapsules (A 8 wt% and B 25 wt%), the fungal cells were hardly observed on the specimens (Fig. 1). The infrared spectra of 3D-printed denture resin discs containing microcapsules filled with phytoncide oil A and B were displayed in Fig. 2. The peaks at 1720 cm^−1^ were detected in both specimens with and without phytoncide-filled microcapsules. The degree of conversion (DC) of 3D-printed denture base specimen with no microcapsules was 43.60 ± 13.83%. All the specimens with microcapsules showed significantly lower DC values compared with those without (all, P = 0.001). For phytoncide type A, the DC values were 14.56 ± 6.72% for 6 wt% and 15.66 ± 8.39% for 8 wt%, respectively. For phytoncide type B, the DC values were 18.03 ± 20.03% and 12.14 ± 3.22% for 15 wt% and 25 wt%, respectively. The specimens from the groups with phytoncide-filled microcapsules, except those of A 8 wt% group, showed significantly lower microhardness and elastic modulus values than the control group (all, P = 0.001, Fig. 3).

**Fig. 4:**
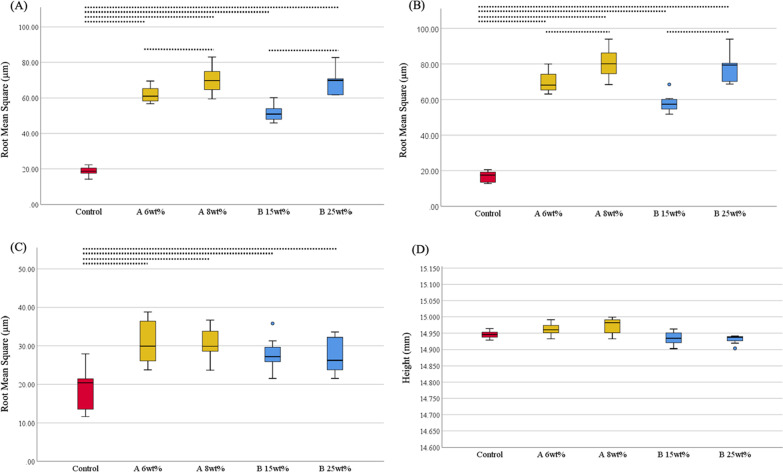
Root mean square (RMS, µm) values and total height (mm) of 3D-printed tower-shaped denture base specimens. **A** Whole surface of specimen; **B** Side surface of specimen; **C** Upper surface of specimen; **D** Total height of specimen. Statistically significant differences among groups were marked with black dotted lines (P < 0.05). Control: no microcapsule; **A**: phytoncide A-filled microcapsule, 6 and 8 wt%; **B**: phytoncide B-filled microcapsule, 15 and 25 wt%

**Fig. 5:**
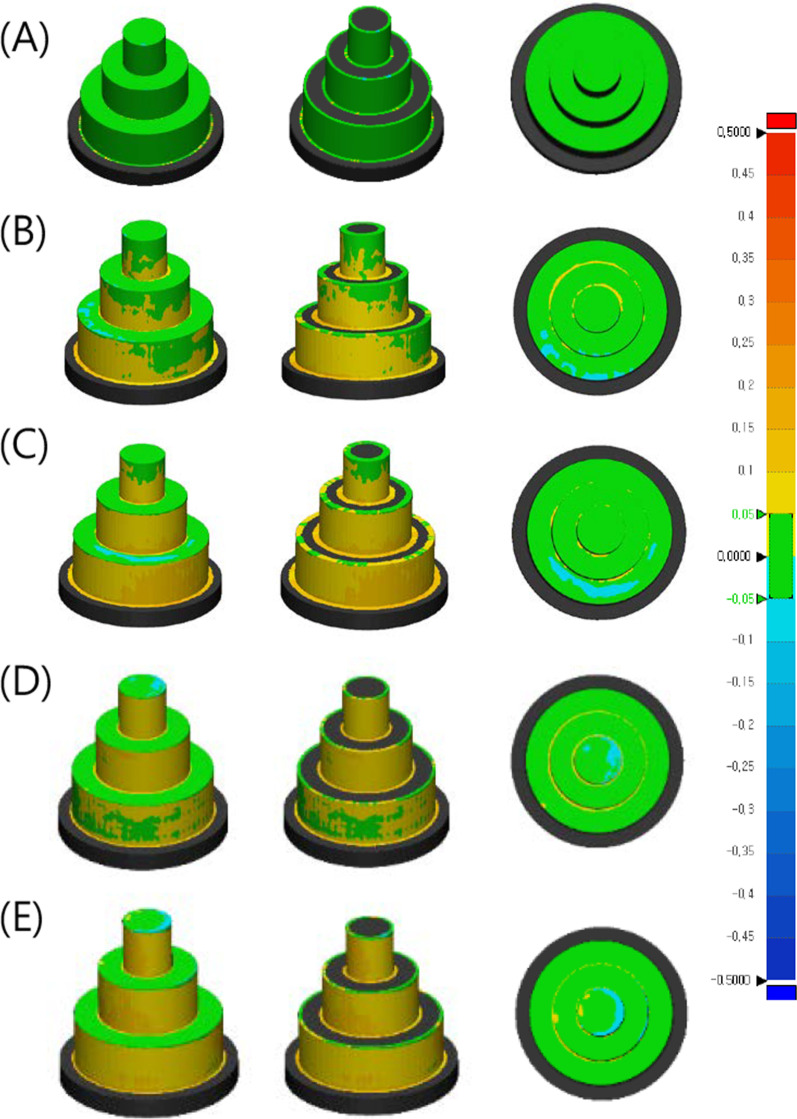
Color deviation maps of 3D-printed tower-shaped denture base specimens. Left to right: whole surface, side surface, and upper surface. **A** Control; **B** phytoncide A-filled microcapsule, 6 wt%; **C** phytoncide A-filled microcapsule, 8 wt%; **D** phytoncide B-filled microcapsule, 15 wt%; **E** phytoncide B-filled microcapsule, 25 wt%. Nominal deviation is ± 50 mm and critical deviation is ± 500 mm

For the whole, upper, and side surfaces of the tower-shaped denture base resin specimens, all the groups with phytoncide-filled microcapsules (A 6 wt%, A 8 wt%, B 15 wt%, and B 25 wt%) showed statistically significant differences (all, P = 0.001) in the mean RMS values from the control group (Figs. 4 and 5). For the whole and side surfaces, the specimens with maximum effective concentration of microcapsules showed significantly higher mean RMS values than those with minimum effective concentration of microcapsules (all, P = 0.001). In terms of total height of the 3D-printed specimens, no statistically significant difference was found between the groups with microcapsules and the control group (all, P > 0.05).

**Fig. 6:**
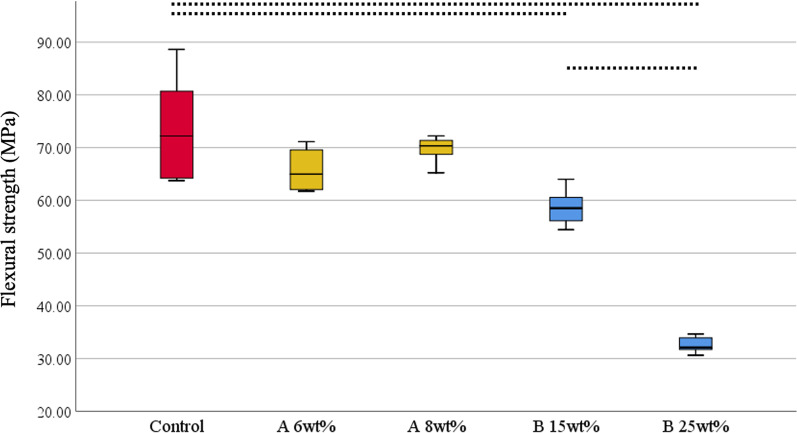
Flexural strength values (MPa) of bar-shaped 3D-printed denture base resin specimens. Statistically significant differences among groups were marked with black dotted lines (P < 0.05). Control: no microcapsule; **A**: phytoncide A-filled microcapsule, 6 and 8 wt%; **B**: phytoncide B-filled microcapsule, 15 and 25 wt%

**Fig. 7:**
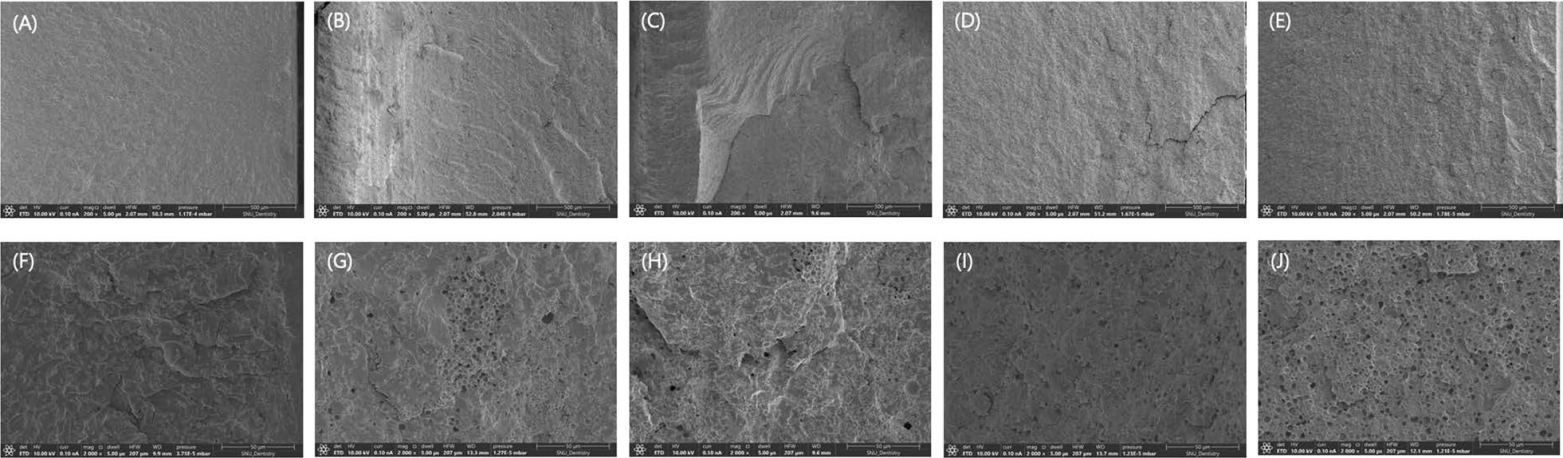
Representative images of microscopic examination of fractured 3D-printed bar-shaped denture base specimens. **A** and **F** Control (× 200 and × 2000, respectively); **B** and **G** phytoncide A-filled microcapsule, 6 wt% (× 200 and × 2000, respectively); **C** and **H** phytoncide A-filled microcapsule, 8 wt% (× 200 and × 2000, respectively); **D** and **I** phytoncide B-filled microcapsule, 15 wt%, (× 200 and × 2000, respectively); **E** and **J** phytoncide B-filled microcapsule, 25 wt% (× 200 and × 2000, respectively). **A** and **F** showed primarily brittle fracture pattern, with smooth surface and river lines (or chevron marks) due to rapid cracks. **B** and **G** showed the mode of intermediate fracture. **C** and **H** showed rough surface revealing ductile fracture, with irregular and jagged areas. **D**, **I**, **E**, and **J** showed typical pattern of intermediate fracture. Most of the incorporated microcapsules were evenly distributed over the fractured surfaces of 3D-printed specimens

The mean flexural strength values of the specimens with phytoncide A-filled microcapsule (both A 6 wt% and A 8 wt%) were higher than 65 MPa, not statistically different from those of the control group (all, P > 0.05, Fig. 6). However, the groups with phytoncide B-filled microcapsules (both B 15 wt% and B 25 wt%) showed significantly lower values than the control (all, P = 0.001). Especially, the mean flexural strength value of B 15 wt% group was significantly higher than that of B 25 wt% group (P = 0.027). The microscopic examination by FE-SEM revealed that the specimens from control group showed primarily brittle fracture patterns, with compact and relatively smooth surfaces due to rapid cracks, while the groups with microcapsules showed patterns of intermediate or ductile fracture, with irregular, rough, and jagged areas (Fig. 7). Most of the incorporated microcapsules were evenly distributed over the fractured surfaces of 3D-printed specimens.

## Discussion

Based on the findings of this study, the null hypothesis was rejected, showing the effect of phytoncide-filled microcapsules with antifungal activity on the microhardness, dimensional accuracy, and flexural strength of the 3D-printed denture base. Generally, the hardness refers to the degree of material resistance to plastic deformation when an indentation load is applied, and is measured as an indicator of resistance to scratching [38]. The results of the current study may have indicated that the 3D-printed denture base containing phytochemical-filled microcapsules could be slightly less resistant to scratching. In addition, the elastic modulus indicates the rigidity of the material, which can minimize the chances of deformation caused by applied stress [39, 40]. In this study, the inclusion of phytochemical-filled microcapsule may have reduced the stiffness of the 3D-printed denture base. However, due to the differences in the evaluation methods, manufacturing parameters, and testing environment, the microhardness evaluated in this study cannot be directly compared to the clinical requirements of denture resin.

The analysis of dimensional accuracy revealed that the phytoncide-filled microcapsule may have affected the deviation on the X–Y plane of 3D-printed denture base, while it had a little or no effect on the deviation to the Z-axis. The tendency may be resulted from the presence of microcapsules as well as the characteristics of light source of DLP technique, led to polymerizing larger area on the X–Y plane than the actual projected area [35]. In the DLP method, the light scattering can make the area to be polymerized larger than the area where the light was projected [41]. However, the clinically acceptable displacement between the intaglio surface of the denture base and the ridge surface was reported to be within 300 µm, as the oral mucosa subsides by that amount during the mastication [9]. Since the mean RMS values from all the groups were lower than 100 µm, the 3D-printed denture base with phytochemical-filled microcapsules could be determined as clinically acceptable.

In this study, no statistical difference in the flexural strength values could be found between the specimens with phytoncide A-filled microcapsules and those of the control group, higher than 65 MPa. However, the inclusion of phytoncide B-filled microcapsules significantly reduced the flexural strength of the 3D-printed denture resin. Based on the previous in-vitro study [34], the overall particle size of phytoncide A-filled microcapsule (1–7 µm) was different from that of phytoncide B-filled microcapsules (5 µm). This may have affected differently on the flexural strength of the 3D-printed denture base, although further studies are required. In fact, mixing microcapsules into the heat-polymerized denture base was reported to reduce the flexural strength [27]. When polycaprolactone microcapsules containing antifungal agents were mixed with polymethacrylate and 3D-printed, the flexural strength of the printed objects was reduced by 35% compared to those made by the conventional process [42]. The microcapsules act as porosities within the denture base, which may have reduced the flexural strength of the specimens. In contrast with the specimens with no microcapsules, microscopic examinations with FE-SEM revealed that the fractured surfaces of the specimens with microcapsules showed intermediate or ductile patterns rather than brittle patterns. Some extent of plastic deformation might have occurred in those specimens, in accordance with the results of microhardness test, slightly less resistant to deformation than the control group. Based on the DC values in this study, the microcapsule may have increased the diffusion or scattering of light and resulted in poor polymerization of the residual monomer, as reported in the previous studies [43, 44]. Interestingly, although the DC was significantly decreased in all the groups with microcapsules compared with the control group, the mean flexural strength value of the control group was not significantly different from those of the groups of phytoncide A-filled microcapsules. Therefore, further studies are required to elucidate the reaction mechanism of microcapsules in terms of type of phytoncide oil extract in the mixture of 3D printable resin. Moreover, to increase the mechanical strength of 3D-printed object, an experimental approach such as adding ceramic-based fillers including silicon dioxide nanoparticles could be applied [45].

There are some limitations of this in-vitro study. First, there was no standardized protocol to evaluate the dimensional accuracy of the dental 3D-printed objects, so this research was conducted using a simplified geometric shape considering the characteristics of light source of a DLP-based printer. Second, numerous parameters in digital light processing such as layer thickness, light intensity, build orientation, and post-processing time, which may affect the mechanical properties or surface qualities of 3D-printed objects, were not evaluated. Third, the effect of thermal cycling or water storage on the mechanical properties of the 3D-printed resin materials were not tested. Lastly, further studies are required to evaluate the feasibility of 3D-printed denture base with phytoncide-filled microcapsules in the clinically simulated situations, considering the disinfection protocols as well as daily cleansing methods.

## Conclusion

Within the limitations of this in-vitro study, the denture base resin material with antifungal activity on *C. albicans* could be manufactured by micro-encapsulation of phytoncide oil and digital light processing technique. Considering the maximum and minimum effective concentrations, the DLP-generated denture base containing 6 wt% concentration of phytoncide A-filled microcapsules was determined as the most acceptable conditions for clinical use, in terms of the antifungal activity, dimensional accuracy, and mechanical properties.

## Data Availability

The datasets generated during and/or analysed during the current study are available from the corresponding author on reasonable request.
